# Comparing in-Person and Telepractice Service Delivery for Spoken Language Production and Comprehension Using the National Outcomes Measurement System

**DOI:** 10.5195/ijt.2021.6373

**Published:** 2021-06-22

**Authors:** Imran Musaji, Blake Roth, Kathy Coufal, Douglas F. Parham, Trisha L. Self

**Affiliations:** 1 Department of Communication Sciences and Disorders, College of Health Professions, Wichita State University, Wichita, Kansas, USA; 2 Department of Communication Sciences and Disorders, Fort Hays State University, Hays, Kansas, USA; 3 Department of Special Education and Communication Disorders, College of Education, Health, and Human Sciences, University of Nebraska at Omaha, Omaha, Nebraska, USA

**Keywords:** NOMS, Outcome measures, School-age Language Disorders, Telepractice

## Abstract

The American Speech-Language-Hearing Association (ASHA) developed the National Outcomes Measurement System for aggregating standardized patient outcomes. Outcomes are standardized using Functional Communication Measures (FCM), scales designed to describe communicative function across specific areas of clinical need. This investigation compared in-person and telepractice service delivery for children in elementary school settings who received treatment targeting the FCM categories of either “spoken language production” or “spoken language comprehension.” De-identified cases were secured from ASHA's NOMS database and the database of a private e-learning provider that implemented the NOMS format. There were minimal significant differences in the median change scores between the traditional and telepractice interventions. These results support comparable treatment outcomes between in-person service delivery and telepractice for treatment of children exhibiting impaired spoken language production or spoken language comprehension in an elementary school setting.

In the field of communication sciences and disorders, there is a well-researched national provider shortage exacerbated by limited openings in graduate training programs, limited opportunities for clinical placements, and inadequate geographic distribution of training and services (Wise et al., 2010). Additionally, the lack of licensing portability across states, despite national accreditation standards and certification requirements, has impeded widespread adoption of telepractice services (Jakubowitz, 2011). It is well-known that delayed or lack of services negatively impacts young and school-age children's development and education (McGill et al., 2020). Although telepractice has been proposed as a potential means of reaching unserved or underserved populations (Doarn et al., 2010), the approach remains underutilized (Lowman & Kleinert, 2017). Prior to COVID-19, regulatory restrictions and reimbursement were key barriers to implementation (Molini-Avejones et al., 2015). Post COVID-19, the primary remaining barrier appears to be the concern that telepractice services may prove inherently less effective than traditional in-person service delivery. Evidence addressing this concern remains limited.

The American Speech-Language-Hearing Association (ASHA) has defined telepractice as the application of telecommunications technology in delivering both audiology and speech-language pathology services by connecting clinician to client or clinician to clinician for assessing, treating, and consulting. The term “telepractice” is preferred over alternate terminology (e.g., eHealth, telehealth, telemedicine, teletherapy) because it is inclusive of both the medical and educational domains. As a result of COVID-19, telepractice has become a more prevalent approach to ensuring the continuity of speech-language pathology services. The most commonly provided services target intelligibility, speech-sound production (i.e., articulation and phonology), and early language abilities, including language comprehension and production (Gabel et al., 2013; Short et al., 2016).

In 2018, Coufal and colleagues reported a retrospective study that compared Functional Communication Measure (FCM) scores after traditional and telepractice services targeting “speech-sound production” and found no significant differences. FCMs are scales designed as part of the National Outcomes Measurement System (NOMS) to allow a standardized format for clinical observations across various communicative functions. The current study, which addresses two other FCM categories (e.g., “spoken language production” and “spoken language comprehensions”), expands the evidence comparing in-person service delivery with telepractice service delivery in elementary school settings using larger sample sizes based on our authors' collective experience in this area.

## LITERATURE REVIEW

Studies involving school-aged children reported that telepractice services are effective, reliable, and efficient (Boisvert & Hall, 2019; Coufal et al., 2018; Grogan-Johnson et al., 2010; Grogan-Johnson et al., 2011; Short et al., 2016). Coufal and colleagues (2018) specifically looked at speech-sound production outcome measures between traditional speech-language pathology service delivery and telepractice. They found no significant difference in treatment outcomes, which supports telepractice as an effective service delivery method. Prior to the work of Coufal et al. (2018), Grogan-Johnson et al. (2011) found similar results, that students in elementary school working on speech-sound production made comparable gains in both traditional treatment and telepractice modalities. Short and colleagues (2016) looked at the effectiveness of a speech and language telepractice program for grades PreK-12 as measured by FCM improvement and time in treatment per week. During the 2012-2013 academic year, 87% of students working on speech-sound production via telepractice services improved by one FCM or more, and 81% of students working on spoken language comprehension and spoken language production via telepractice improved by one FCM level or more. Similarly, throughout the 2013-2014 academic year, 83% of students working on speech-sound production via telepractice services improved by one FCM or more, 79% of students working on spoken language comprehension via telepractice improved by one FCM level or more, and 67% of students working on spoken language production improved by one FCM level or more. In addition to the progress made, students spent less time in treatment sessions per week via telepractice: 54% of students served through telepractice received services for less than 40 minutes per week, whereas 74% of students served through traditional in-person services were seen 42-60 minutes per week. These results support telepractice as equally effective as traditional services over a shorter duration per week.

In the past, some have cited concerns about expense, technical difficulty, and low buy-in as barriers to implementation. Recent studies refute these concerns. Telepractice service delivery modalities are implemented relatively easily and efficiently (Grogan-Johnson et al., 2010; Short et al., 2016). Boisvert and Hall (2019) reported that with appropriate introduction and training, stakeholders adopted telepractice modalities enthusiastically and with positive post-intervention perceptions. Lincoln et al. (2014) interviewed multiple stakeholders, including parents, school administrators, and clinicians in a rural school district to determine views on telepractice for speech-language pathology services, and found uniformly positive assessments. Additionally, Hines et al. (2015) found no negative effects of telepractice on clinicians' and pediatric clients' rapport. While these findings are encouraging, widespread implementation requires that research be expanded beyond the pilot and exploratory phases, include larger samples sizes (Coufal et al., 2018; Grogan-Johnson et al., 2010), and utilize objective outcome measures relevant to clinical translation (Coufal et al., 2018; Musaji et al., 2019). These needs are directly addressed by the NOMS system, which aggregates national outcomes data across FCM categories to enable research into the efficacy of various clinical programs.

A systematic literature search of studies published between 2010 and 2020 was conducted to identify currently available evidence comparing outcomes of telepractice versus traditional in-person intervention specifically targeting either language comprehension or language production at the elementary school level within the American education system. Only one peer-reviewed study addressed such a comparison. Gabel et al. (2013) compared telepractice and in-person outcomes for the four most frequently occurring FCM categories (i.e., speech-sound production, spoken language production, spoken language comprehension, and intelligibility). They reported similar functional improvement for telepractice and in-person interventions targeting intelligibility or speech-sound production.

The primary limitation of this study was the smaller sample-size for the telepractice cohort (n=71) compared with a larger NOMS dataset (n=5,332). This difference between sample sizes reduces the statistical power of any comparisons and increases the chance of Type I error (Rusticus & Lovato, 2014). Additionally, because the smaller cohort of telepractice students was distributed among four FCMs and six severity subgroups, there were very few individuals representing telepractice intervention for each specific level of comparison.

Perhaps because of these limitations, there were some unexpected variations in their results. For example, for the FCM “spoken language production” telepractice was less effective than in-person intervention (56.4% of participants improving vs. 71.1% of participants improving, respectively). For the FCM “spoken language comprehension” the outcomes varied unpredictably by severity subgroup. However, on average, there was no statistically significant difference in outcomes between the two groups, supporting the feasibility of telepractice provision of services.

It is important to acknowledge that comparisons of telepractice and traditional modalities are being conducted for a variety of other intervention targets. There were multiple studies that focused on coaching models for treatment, often for early-language intervention (Akemoglu et al., 2020; Baharav & Reiser, 2020; Behl et al., 2017). These tended to report similar outcomes or improved outcomes when using telepractice compared with in-person delivery.

Also notable, international researchers are asking many similar questions (Cangi & Toğram, 2020; Fairweather et al., 2016; McCarthy et al., 2012; Rao et al., 2018; Shprintzen & Golding-Kushner, 2012). In general, these studies have found telepractice outcomes consistent with or better than in-person delivery. However, national variation in scope of practice and training for speech-language pathologists, different barriers to implementation (e.g., regulatory, reimbursement, cultural), and different outcome measures all make international comparisons vulnerable to multiple confounds. More specifically, the NOMS system explicitly measures functional outcomes within the American education system. For this reason, two articles outside the United States were excluded. Any articles targeting individuals with English as a second language were also excluded.

## PURPOSE

The available evidence is limited but appears to support the use of telepractice as comparable to traditional speech therapy delivery methods with children exhibiting language delays or impairments. This study sought to expand the evidence base comparing telepractice with traditional in-person service delivery by including a larger sample of children served in school-based settings and use of a common metric for comparisons (FCMs), which has been a limitation in many other studies. Two categories of FCM (spoken language production and spoken language comprehension) underrepresented in the extant literature were targeted. A major limitation of the ASHA NOMS database from 1999-2018 was that it only included records from in-person service delivery. A new NOMS database launched in 2020 that does include the ability to report telepractice outcomes, but limited data is available at this time. A search of telepractice providers found only one national school-based provider (i.e., PresenceLearning) whose clinicians participated in the ASHA NOMS training, and utilized the NOMS FCMs and data conventions. PresenceLearning is a private provider that collaborates with K-12 public schools nationwide to provide online special education related services. The investigators used complete data, with permission, from the retrospective ASHA NOMS database and the PresenceLearning database. Data from both databases were de-identified and provided for research and reporting without any conditions on use.

This study addresses limitations of prior studies by including a larger telepractice cohort for comparison; by filtering the data to remove potential confounds such as differences in age, intervention targets, initial functional levels, and differences in duration of treatment; and by utilizing a data-standardized format. Notably, the effects of alternating treatment sequences, which had been a condition of earlier research, do not influence performance outcomes reported in the study by Coufal et al. (2018) and the current investigation.

Speech-language pathologists collected all data in an elementary school environment. The controlled setting allowed the investigators to report on treatment provided in functional contexts. By collecting data from established providers of each service modality, the study was more naturalistic and thus able to mitigate the potential confounds of variable clinician experience, novel technology, and disturbance of the school day's typical routine. This study was approved by the local Institutional Review Board. Both ASHA and the private e-learning provider provided permission to use these datasets unconditionally. ASHA provided NOMS data including the time span from 1999 to 2010. PresenceLearning provided data from 2013 to 2014. The same datasets were utilized in the initial study comparing telepractice and in-person outcomes targeting the FCM speech-sound production (Coufal et al., 2018). To date we are unaware of any alternative data sets that would allow comparison of telepractice and in-person services, but it should be noted that the new ASHA NOMS system that has just been launched includes the ability to upload and code outcomes from telepractice providers. This change will hopefully allow new and updated outcome data to be used for future studies.

## METHOD

## NATIONAL OUTCOMES MEASUREMENT SYSTEM (NOMS)

The NOMS FCMs provided the common metric used to compare the intervention provided through traditional services and those provided via telepractice. NOMS is a voluntary data collection system developed by the American Speech-Language-Hearing Association (ASHA) to standardize and quantify speech-language intervention outcomes. NOMS uses Functional Communication Measures (FCMs), which are “a series of disorder-specific, seven-point rating scales designed to describe the change in an individual's functional communication and/or swallowing ability over time” (ASHA, 2015, para. 5). Clients are assigned a pre- and post-treatment score (using a 1-7-point scale, with lower scores representing less functional ability), and change scores are calculated using these measures. The NOMS rubrics for scoring the “spoken language production” and “spoken language comprehension” FCMs are provided in Appendix A. ASHA's pediatric NOMS have been previously researched for validity and reliability (Mullen and Schooling, 2010; Gallagher et al., 1998; National Treatment Outcome Data Collection Project, 1997; National Outcomes Measurement System, 1997).

The data for NOMS/FCM was collected within public schools throughout the United States. Clinicians contributing to either database completed ASHAs standardized NOMS User Training program. Participating clinicians were all trained to use the NOMS/FCM scoring system, but otherwise had varying levels of expertise and were providing treatment to students of varying demographics. Therefore, the FCMs offer a standardized scale that can be used to compare traditional and telepractice intervention outcomes (Mullen & Schooling, 2010).

## NOMS DATA

ASHA and the telepractice providers provided the data used in this study in an Excel database format. Providers de-identified all data before it was sent to the investigators. Both databases included all NOMS data in its entirety for the requested period. At no time during this study did ASHA personnel or the telepractice provider have access to the data files or input into the data that were selected for this study.

## CASE SELECTION

For both categories (spoken language production and spoken language comprehension) reported scores of children who were provided intervention in a school setting from any geographic region within the U.S and met the following criteria were included in the analysis: (1) the children received treatment exclusively for only one or the other category, (2) were between six and nine years of age, and (3) had treatment that lasted between four to nine months. Four to nine months is consistent with service delivery in the public school systems, and prior studies have suggested this duration corresponds to functional improvement of approximately 1 FCM with traditional in-person service delivery (Jacoby et al., 2002). Table 1 provides the distribution of cases for both the spoken language production and spoken language comprehension categories, across all inclusion criteria, for both the ASHA and telepractice NOMS databases.

**Table 1:** Number of Cases per Inclusion Criteria

Inclusion criteria	ASHA NOMS Group	Telepractice Group
Spoken language production		
1. Clients received treatment exclusively for spoken language production	6058	2152
2. Clients met criterion 1 and were between 6.0 to 9.0 years of age	3994	645
3. Clients met criteria 1 and 2 and had treatments lasting between 4 and 9 months	1213	408
Spoken language comprehension		
1. Clients received treatment exclusively for spoken language comprehension	4691	1712
2. Clients met criterion 1 and were between 6.0 to 9.0 years of age	3000	424
3. Clients met criteria 1 and 2 and had treatments lasting between 4 and 9 months	946	254

## ANALYSIS

This study was organized as a retrospective three-factor analysis. The key factors were intervention target (i.e., spoken language production or spoken language comprehension), intervention approach (i.e., in-person or telepractice), and the severity subgroup as determined by the initial FCM level prior to treatment. It was essential to clean and prepare the data to ensure it was comparable across each factor.

First, each intervention target group's data was filtered using the age (between six and nine years) and length of treatment (between four and nine months) criteria to ensure any comparisons were similar across these two variables. Note that discrepancies in how age data were entered between the two databases prevented more granular comparisons. The in-person database reported age as an integer (i.e., 6, 7, 8, and 9), while the telepractice database precisely calculated age at the time of treatment. Attempting to compare “six-year-olds” between groups would result in overly large confidence intervals. Instead, the comparison focused on an age range relevant to the FCMs of spoken language production and spoken language comprehension (between six and nine years of age). Similarly, there was a discrepancy in how the length of treatment was reported in the two databases. The in-person database reported the number of days of skilled service, while the telepractice database calculated the total duration from start of treatment to end of treatment. A broad range of four to nine months encompassed the range of duration for most in-school service delivery across both methods of calculation and thus avoided the discrepancy. Refer to Table 1 for the cases eliminated with each inclusion criteria. This step was essential to ensure comparability of data and elimination of potential confounds, however many cases failed to meet the inclusion criteria. Ensuring standardized formats for data collection of both in-person and telepractice service delivery should be a priority for future clinicians and researchers.

Second, data were sorted into severity subgroups for each FCM category. Individuals from each database were sorted into a severity subgroup depending on their initial FCM score ranging from “1” to “5.” It is common knowledge that language development does not follow a linear trajectory, particularly during treatment. Initial FCM scores provided a common starting point for group comparisons. The ratio of in-person cases to telepractice cases ranged from approximately 2:1 to approximately 4:1 for spoken language production and approximately 2:1 to approximately 6:1 for spoken language comprehension.

Third, the dependent variable (i.e., the median change between the initial and final FCM scores) was calculated for each subgroup. Because the FCMs rank performance on seven-point scales, the range of change scores is limited. Median scores are the most appropriate measure of central tendency for range-limited variables, and a Mann-Whitney U Test is the most appropriate procedure for comparing median scores of ordinal data. Mann-Whitney U Tests were used to test for differences between the medians of the two groups.

## RESULTS

Descriptive statistics for both age and length of treatment are presented for spoken language production in Table 2, and for spoken language comprehension in Table 3. For spoken-language production, the in-person data demonstrated a moderate positive skewness, while telepractice results were approximately symmetrical. Kurtosis values less than one for both cohorts indicated nonnormal light-tailed distributions (Hair et al., 2017, p. 61). For spoken-language comprehension, both groups were distributed symmetrically and demonstrated nonnormal light-tailed distributions. Considering the nonnormal distributions and that NOMS FCM results are rank-ordered (ordinal) data, a non-parametric analysis was indicated.

**Table 2:** Distributional Data for Age and Length of Treatment Criteria between Groups for Spoken Language Production

	ASHA NOMS Group (*n* = 1,214)	Telepractice Group (*n* = 408)	Absolute difference (%)
Age between six and nine years (years)			
*M*	7.44	7.90	0.46 (5.82)
*SD*	1.08	0.82	0.26 (31.71)
Skewness	0.58	-0.25	--
Kurtosis	-1.27	-1.06	--
*Mdn*	7.00	7.75	0.75 (9.68)
Range	3.00	2.94	0.06 (2.04)
Minimum	6.00	6.03	0.03 (0.50)
Maximum	9.00	8.98	0.02 (0.22)
Length of treatment between four and nine months (days)			
*M*	191.67	206.90	15.23 (7.36)
*SD*	41.68	41.30	0.38 (0.92)
Skewness	0.02	-0.65	--
Kurtosis	-1.16	-0.80	--
*Mdn*	191.00	222.64	31.64 (14.21)
Range	150.00	149.04	0.96 (0.64)
Minimum	120.00	120.53	0.53 (0.44)
Maximum	270.00	269.56	0.44 (0.16)

**Table 3:** Distributional Data for Age and Length of Treatment Criteria between Groups for Spoken Language Comprehension

	ASHA NOMS Group (*n* = 1,214)	Telepractice Group (*n* = 408)	Absolute difference (%)
Age between six and nine years (years)			
*M*	7.48	7.70	0.22 (2.86)
*SD*	1.08	0.89	0.19 (21.35)
Skewness	0.03	-0.38	--
Kurtosis	-1.26	-1.10	--
*Mdn*	7.00	7.88	0.88 (11.17)
Range	3.00	2.96	0.04 (1.35)
Minimum	6.00	6.03	0.03 (0.50)
Maximum	9.00	8.99	0.01 (0.11)
Length of treatment between four and nine months (days)			
*M*	190.03	207.74	17.71 (8.53)
*SD*	41.54	40.80	0.74 (1.81)
Skewness	0.04	-0.63	--
Kurtosis	−1.18	−0.84	–
*Mdn*	189.00	224.65	35.65 (15.87)
Range	150.00	146.98	3.02 (2.05)
Minimum	120.00	122.58	2.58 (2.10)
Maximum	270.00	269.56	0.44 (0.16)

Descriptive analysis of each severity subgroup (differentiated by initial FCM score) is presented in Table 4 for each intervention target (i.e., spoken-language production or spoken-language comprehension). The medians and interquartile ranges were similar for all subgroups. It is important to note that for median-appropriate distributions, the range, minimum scores, and maximum scores are the least informative descriptive measures, given that a small number of individual scores can unduly influence their interrelationship.

**Table 4:** Distributional Data for Change Scores Associated with Each Severity Subgroup by Intervention Modality

	ASHA NOMS Group	Telepractice Group	Absolute difference
Initial FCM score = 1			
*n*	26	15	--
*Mdn*	1	1	0
Interquartile range	1	1	0
Range	5	6	1
Minimum	0	0	0
Maximum	5	6	1
Initial FCM score = 2			
*n*	172	79	--
*Mdn*	1	1	0
Interquartile range	1	1	0
Range	5	4	1
Minimum	0	0	0
Maximum	5	4	1
Initial FCM score = 3			
*n*	420	142	--
*Mdn*	1	1	0
Interquartile range	1	0	1
Range	6	3	3
Minimum	-2	0	2
Maximum	4	3	1
Initial FCM score = 4			
*n*	336	105	--
*Mdn*	1	1	0
Interquartile range	1	1	0
Range	5	3	2
Minimum	-2	0	2
Maximum	3	3	0
Initial FCM score = 5			
*n*	188	47	--
*Mdn*	1	1	0
Interquartile range	2	1	1
Range	3	2	1
Minimum	-1	0	1
Maximum	2	2	0

To assess if there was a meaningful difference in the median change of FCM scores between the in-person intervention versus the telepractice intervention, non-parametric Mann-Whitney U Tests were utilized. The results are presented in Table 5. Where significant median differences were observed, an effect size statistic (η^2^) was calculated to determine the approximate percentage of variation explained by the difference in interventions (Fritz et al., 2012).

**Table 5:** Median Difference Scores for Spoken Language Production and Spoken Language Comprehension by Initial FCM Score

Initial score	ASHA NOMS Group		Telepractice Group					
	*n*	*Md*	*n*	*Md*	*Mann-Whitney U*	*p*	*η^2^*
Spoken language production							
1	26	1.0	15	1.0	187.0	.817	
2	172	1.0	79	1.0	5330.0	.003*	.035
3	420	1.0	142	1.0	24759.0	.001*	.019
4	336	1.0	105	1.0	17046.0	.576	
5	188	1.0	47	1.0	3919.0	.203	
Spoken language comprehension							
1	16	1.0	7	1.0	45.0	.402	
2	124	1.0	34	1.0	1766.0	.124	
3	323	1.0	94	1.0	13032.5	.023*	.012
4	262	1.0	88	1.0	10876.0	.394	
5	161	1.0	25	1.0	1860.0	.512	

*Note.* An estimate of effect size (η^2^) is provided only for those severity subgroups that demonstrated significant median differences *p<.05.

For spoken-language production, Mann-Whitney tests indicated severity subgroup 3 (i.e., initial FCM=3) benefitted slightly more from telepractice intervention than in-person, U = 13,032.5, p = .023, η^2^ = .012. Otherwise, no significant median differences were observed between telepractice and in-person outcomes for any other subgroups. For spoken-language comprehension, Mann-Whitney tests indicated severity subgroup 2 and 3 (i.e., individuals with initial scores of FCM 2 or 3) benefitted slightly more from telepractice intervention than in-person, U_subgroup2_ = 5,330.0, p = .003, η^2^ = .019; U_subgroup3_ = 24,759.0, p = .001, η^2^ = .035. Otherwise, no significant median differences were observed between telepractice and in-person outcomes for any other subgroups. Across all subgroups, the median change was one functional level as a result of the therapeutic intervention.

## DISCUSSION

The purpose of this study was to compare outcomes for students in an elementary-school setting receiving either in-person or telepractice services targeting the FCM categories of “spoken language production” or “spoken language comprehension.” Children receiving speech therapy targeting multiple FCM categories were excluded to control for potential confounding treatment interactions. Data were filtered to control for age, length of therapy, and severity subgroup (i.e., FCM score at the initiation of treatment).

Both cohorts demonstrated positive outcomes as a result of treatment. The results of our analysis of these select cohorts suggest that there is either no significant difference in treatment outcomes between traditional in-person interventions or, in a few instances, a small improvement in outcomes with telepractice services. The significant differences observed for three of the ten severity subgroup comparisons accounted for between 1 and 3 percent of the observed variation in FCM scores for children participating in telepractice services. These results support and expand the literature supporting the use of telepractice as effective service delivery.

These findings strengthen those reported by similar studies (Gabel et al., 2013; Grogan-Johnson et al., 2011) with the added robustness of expanded sample size and added controls against confounding interactions. These findings are also consistent with Coufal and colleagues' (2018) examination of telepractice outcomes in the FCM category “speech-sound production.” Finally, these findings support the emerging broader consensus that telepractice, across a wide spectrum of professions and interventions, is comparable with in-person delivery.

Given the apparent equivalency of telepractice and in-person delivery modalities for the two examined FCM categories, the problem of access to qualified service providers and quality interventions for children with impairments in these categories may be ameliorated through telepractice. According to ASHA (2019), between 40 percent to 60 percent of children treated by school-based speech-language pathologists have spoken language production and spoken language comprehension impairments. The findings of this retrospective study provide evidence that children presenting with language impairments improve their communication abilities when services are provided via telepractice. Given these findings, coupled with other sources of evidence, professionals and consumers can be confident that a gap in current practices can be addressed as an increasing number of telepractice providers work within schools.

## IMPLICATIONS

Existing outcome metrics (such as NOMS) appear appropriate measures for telepractice, and by using them, it is possible to directly compare the two modes of service delivery. This study provides further evidence that for some of the highest frequency FCM categories, there are negligible differences in outcomes between in-person therapy and therapy via telepractice.

Service delivery targeting the FCMs “spoken language production” or “spoken language comprehension” for a select group of children resulted in equivalent outcomes regardless of whether a telepractice or in-person modality was used. While telepractice might not be appropriate for every client, the results of this comparison further support the potential for telepractice as an effective mode of service delivery. This alternative provides (a) more flexibility in therapy delivery, (b) opportunity for dispersal of school caseloads, and (c) increased potential for data sharing among clinicians utilizing telepractice.

## LIMITATIONS

This study compared de-identified data from the ASHA NOMS database and an independent telepractice provider's NOMS database. The use of large datasets with systematically formatted responses facilitates improved inter-reporter reliability and larger sample sizes providing for more robust statistical conclusions. However, significant granularity in data is lost when using such a system. Additional quantitative and qualitative justifications for individual and cohort change scores could not be provided due to the lack of variables provided via the data sets (e.g., gender, race, ethnicity, socioeconomic status, or compliance during therapy).

Further, the duration and frequency of treatment sessions were not specified. The only determinable information available is that the students received at least one treatment session per week during the time the initial FCM score was assigned and the final FCM determination. Thus, age and duration/frequency of intervention could not be factored into the analysis. Rather, age and length of treatment were used as inclusion criteria to establish comparable groups between the two datasets. Exploring variables related to the SLPs who provided treatment would have been of interest to the investigators. Unfortunately, neither dataset provided information relative to the clinicians' professional experience, areas of expertise, and/or treatment strategies used. Had this information been available, it may have been possible to determine why scores changed under certain conditions.

Information on variables impacting inclusion in either database was not available, except for categories specifically defined by the NOMS system. However, NOMS training explicitly states that data for any child for whom services are provided should be entered into the system. It is possible that some students were selectively included or excluded from receiving telepractice services based on accessibility variables. This is beyond the scope of this study but is a consideration for future research.

## FUTURE DIRECTIONS

From a translation science perspective, this study might be categorized as a T2 study along the implementation continuum (Musaji et al., 2019). It compares patient outcomes for established interventions to demonstrate clinical efficacy and potential best-practices. There are many other potential research targets to be addressed at this stage. While this study suggests that telepractice intervention can be equivalent to in-person intervention for specific pediatric FCM categories, it will be important to determine if these results remain consistent across other pediatric and adult FCMs. Any future retrospective studies must be careful to isolate proposed explanatory variables as has been done here. Additionally, replication of this and similar studies will be needed to ensure the findings are consistent across demographics.

To translate T2 findings to clinical practice, either large randomized controlled studies are needed, or large, standardized aggregate data sets are needed to allow for more statistically powerful and granular analyses. Later studies may need to develop more detailed and objective quantitative systems for data collection than NOMS currently provides. Alternatively, research is needed to determine the optimal parameters for telepractice intervention and how much training practitioners need to achieve positive outcomes. Further, studies could be designed to investigate potential socioeconomic barriers to the implementation of telepractice in some districts.

A 2005 systemic review of medical literature by Choudhry and colleagues found that with increasing years of practice, some practitioners provided decreasing quality of care. One proposed explanation for these results is that medical knowledge's doubling time is so fast that the knowledge translation process has not kept up (Densen, 2011). The subfield of telepractice is expanding especially rapidly. Telepractice exists at an intersection of multiple evolving fields (e.g., healthcare, technology, and education), and necessity accelerates this dynamism further. Vigorous research in this area is needed, and efforts to translate these findings to clinical education and practice must follow.
